# A24 INCREASE IN OXIDATIVE STRESS AS MEASURED BY SERUM CYSTEINE-BASED REDOX SENSING, IS ASSOCIATED WITH FUTURE RISK OF CROHN’S DISEASE.

**DOI:** 10.1093/jcag/gwae059.024

**Published:** 2025-02-10

**Authors:** K Mu, C Dang, M Xue, J Shao, S Lee, Q LI, A Griffiths, A Steinhart, L Dieleman, G Aumais, R Panaccione, W Turpin, K Croitoru

**Affiliations:** Department of Medicine, University of Toronto, Toronto, ON, Canada; Department of Medicine, University of Toronto, Toronto, ON, Canada; Sinai Health, Toronto, ON, Canada; Department of Medicine, University of Toronto, Toronto, ON, Canada; Sinai Health, Toronto, ON, Canada; Sinai Health, Toronto, ON, Canada; Sick Kids Foundation, Toronto, ON, Canada; Sinai Health, Toronto, ON, Canada; University of Alberta, Edmonton, AB, Canada; Hopital Maisonneuve-Rosemont, Montreal, QC, Canada; University of Calgary, Calgary, AB, Canada; Sinai Health, Toronto, ON, Canada; Sinai Health, Toronto, ON, Canada

## Abstract

**Background:**

Crohn’s disease (CD) is a gastrointestinal disorder characterized by chronic inflammation. While increased oxidative stress is observed in established CD patients, it remains unknown whether a shift in redox status is present before the diagnosis of CD and whether it is correlated with changes in immune response and microbial composition. Our hypothesis is that oxidative stress plays a role in the development of CD, and it could be detected before the diagnosis of CD.

**Aims:**

We aimed to assess the association between the serum redox state-related indicators, specifically cysteine (reduced form) and cysteine sulfinic acid (oxidized form), quantified by mass spectrometry, with future risk of CD and if this is further correlated with markers of systemic inflammation and alterations in gut microbiota composition.

**Methods:**

In the Genetic Environment Microbial Project, a cohort of first-degree relatives (FDRs) of CD patients was prospectively followed. Among them, we identified subjects who later developed CD, defined as pre-CD (n=69), and matched them at a 1:4 ratio with FDRs who remained disease-free (n=276). Conditional logistic regression assessed the association with future CD. Partial Spearman regression evaluated correlation with systemic inflammation, as indicated by c-reactive protein (CRP), and with changes in gut microbiota composition (determined by fecal 16S rRNA sequencing).

**Results:**

Elevated cysteine was negatively associated with the likelihood of developing CD (Est = -1.18; *p* = 0.02) and negatively correlated with CRP levels (coeff = -1.04; *p* = 0.028). In contrast, cysteine sulfinic acid was positively associated with CD (Est = 1.15; *p* = 0.0004) and positively correlated with CRP levels (coeff = 1.11; *p* = 0.001). Specifically, their association with CD is independent of CRP levels. Three microbial risk taxa, which we previously reported to be associated with the future development of CD, were significantly correlated with cysteine sulfinic acid (*p* = 0.05).

**Conclusions:**

This study is the first to report that when redox status shifts towards oxidative stress, as indicated by the level of cysteine and cysteine sulfinic acid, the likelihood of CD increases. Furthermore, these markers also correlate with early pre-clinical inflammation, as indicated by CRP levels as well as gut microbiota composition, indicating a change of gut microbiota composition when the redox status shifts towards oxidative stress.

**Funding Agencies:**

